# Measuring ethylene in postharvest biology research using the laser-based ETD-300 ethylene detector

**DOI:** 10.1186/s13007-018-0372-x

**Published:** 2018-11-28

**Authors:** Sunny George Gwanpua, Abdul Jabbar, Jeritah Tongonya, Sue Nicholson, Andrew R. East

**Affiliations:** Massey AgriTech Partnership, Massey University, Private Bag 11222, Palmerston North, 4412 New Zealand

**Keywords:** Ethylene production, Operational curves, Continuous flow method, Stop and Flow method, Residence time, Time delay

## Abstract

**Background:**

Ability to measure ethylene is an important aspect of postharvest management, as knowledge of endogenous ethylene production is used in assessing physiological status, while response of crops to exogenous ethylene informs efforts needed to control unwanted ripening. An ethylene monitoring device with a laser-based photoacoustic detector, ETD-300, was recently developed by Sensor Sense B.V., Nijmegen, The Netherlands. In terms of performance, the ETD-300 is superior to all other current ethylene measurement devices, with a sensitivity of 0.3 nL L^−1^, a response time of 5 s, and an ability to monitor ethylene in real time. Although the ETD-300 is relatively easy to operate, the performance and correctness of the data obtained depends on the choice of settings, which depends on the application.

**Results:**

This article provides a description of different ways in which the ETD-300 can be used in postharvest research for monitoring ethylene production and ethylene presence in an environment. We provided guidelines on selecting the appropriate method (Continuous Flow, Stop and Flow, and Sample methods), and operational curves for deciding on suitable combination of free volume, flow rates, and period for the different measurement methods.

**Conclusions:**

Using these guidelines and operational curves, ETD-300 users can considerably reduce the measurement effort by limiting trial and error in establishing appropriate methodologies for their application. The guidelines also comment on accurate use of the ETD-300, as using the inappropriate settings could lead to erroneous measurements. Although these methodologies were developed primarily for postharvest application, they can be applied in other plant science research.

## Background

Ethylene is an important gaseous phytohormone involved in the regulation of growth, development, ripening, and senescence of many fruit and vegetables [1, 2]. In postharvest management of crops, exogenous ethylene has been shown to induce changes in a number of ripening-related quality attributes, such as softening [3–7], changes in peel colour [5–7], increase production of aromatic volatiles [6, 8–10], and increase in soluble solids content [6, 11].

Fruit of climacteric nature produce large amounts of ethylene at the onset of ripening, and initiate endogenous ethylene production in response to application of ethylene [12]. Consequently, ethylene production could be used for certain climacteric fruit as an indicator of ripening progression. For example, internal ethylene concentration or production rate is commonly used as a maturity index to determine commercial harvest dates for apples fruit [13–15].

An important consideration in postharvest management of quality is how produce respond to exogenous ethylene, as this helps to determine the need to prevent unwanted ripening and senescence. A survey conducted to determine the level of ethylene in the atmosphere of fruit and vegetable in holding areas, distribution centres, and supermarket retail stores revealed concentrations of between 0.017 and 0.2 μL L^−1^ [16]. For several decades it was believed that environmental ethylene concentrations > 1 μL L^−1^ are needed to initiate many of the physiological responses that are influenced by ethylene. This was in part driven by the inability of ethylene sensing equipment, mainly gas chromatography (GC), to measure ethylene concentrations of less than 1 μL L^−1^. However, recent advancement in ethylene sensing technologies able to measure much lower concentrations have led to discoveries that ethylene in the nL L^−1^ concentrations are sufficient to influence ripening physiology of certain crops [6, 16–19]. Examples of these recent technologies are the flame ionisation detection (FID) and photoionization detector (PID) for use in GC [20], electrochemical sensors [21], and laser-based photoacoustic detection [22–24]. Of all current ethylene-sensing technologies, the laser-based sensors have the highest sensitivity (below nL L^−1^), fastest response time (seconds), good selectivity and capability of real-time monitoring [25]. Cristescu et al. [25] reviewed current methods for detecting ethylene in plants and demonstrated that laser based sensors, such as the ETD-300 has a sensitivity of about 1 nL L^−1^, while GCs which has traditionally been used to detect ethylene in plant sciences has a sensitivity of between 10 and 100 nL L^−1^. In addition, the ETD-300 has a response time of 5 s, compare to GCs that have response times between 200 and 1000 s, making the ETD-300 suitable for real-time minoring of ethylene. Finally, the ETD-300 has a much higher selectivity than most GCs. The main drawback of the ETD-300 is that it is more expensive than other ethylene detection systems.

The ETD-300 (Sensor Sense B.V., Nijmegen, The Netherlands) is a commercial CO_2_ laser-based photoacoustic ethylene detector that can measure real-time ethylene concentration as low as 0.3 nL L^−1^. The ETD-300 has been used in several postharvest studies where capability to measure low ethylene concentrations was needed (Table 1). The most common application has been to measure ethylene production of crops following different treatments, while a few authors have used the ETD-300 to monitor ethylene concentrations. Beyond postharvest studies, the ETD-300 has been used to measure ethylene production in *Arabidopsis* in a number of fundamental plant biology studies [25–29].

**Table 1:** Review of the use of the laser-based ethylene detector ETD-300 in postharvest biology

Type of measurement	Research summary	References
Ethylene production	Online monitoring of wound-induced ethylene production in fresh-cut endive in response to 1-MCP, AVG and heat shock treatments	[30]
Ethylene production	Real-time measurement of ethylene production in kiwifruit that were bruised, sliced, pricked, or inoculated with *Botrytis cinerea*	[31]
Ethylene production	Investigate the effect of ABA-deficiency in ethylene evolution rate in tomato	[32]
Ethylene production	Demonstrate inhibition of ethylene production in strawberry fruit using a commercial ethylene scavenger	[33]
Ethylene production	Ethylene production during ripening of feijoa as influenced by harvest maturity, storage duration, treatment with 1-MCP, or ethylene	[34]
Ethylene production	Ethylene production of feijoa following pre-harvest application of aminoethoxyvinylglycine	[35]
Ethylene production	Ethylene production during ripening of ‘Kensington Pride’ mango fruit following treatment with 1-MCP and/or ethylene	[36]
Ethylene production	Ethylene production during storage of different gooseberry cultivars	[37]
Ethylene production	Ethylene production of avocado and strawberry during storage following different scheduling of controlled atmosphere	[38]
Ethylene production	Ethylene production of fresh *Piper nigrum* berries following treatment with different doses of UV-C	[39]
Ethylene diffusion	Ethylene diffusion properties of commercial kiwifruit polyliners	[40]
Ethylene concentration	Monitoring ethylene concentration in gas mixes when mixing standard ethylene gases and air to generate ethylene of certain desired concentration	[17, 18]
Ethylene concentration	Ethylene removal rate by vacuum ultraviolet radiation	[41]

While the ETD-300 is relatively easy to use, the performance and efficiency are highly dependent on the choice of settings, which is dependent on what ethylene concentration is to be measured, and the scenario on which the measurement is being utilised (i.e. ethylene production versus environmental monitoring). There is also a learning process to adaptation of the ETD-300 as opposed to Gas Chromatography, as sampling of small volumes (few millilitres) is not possible. The objective of this paper is to provide a guideline on the efficient use of the ETD-300 for measuring ethylene in different systems in postharvest biology research, although the same principles will apply for all plant systems.

## Results and discussion

### Description of the ETD-300

**Fig. 1:**
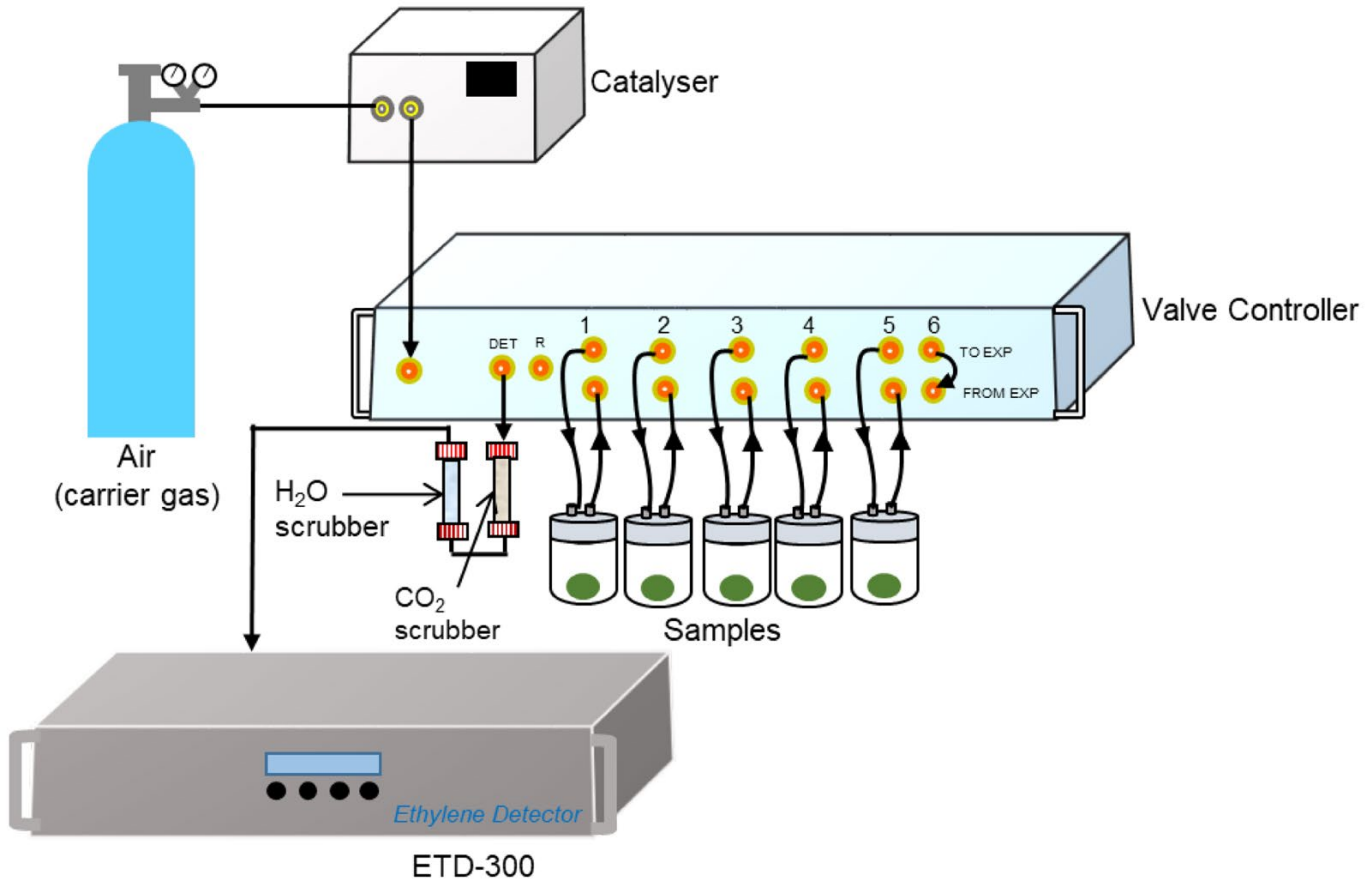
A schematic representation of the ETD-300 set for measurement of ethylene production of different samples, where channel 6 is set up to be a zero calibration value

The ETD-300 detector is often used with a catalyser and one or more valve controllers (Fig. 1). The catalyser removes any hydrocarbon in the carrier gas (usually dry air) enabling a supply of ethylene free gas, while the valve controllers enables automatic sampling and mass flow control. As CO_2_ interferes with the readings of ETD-300, the sample gas is passed through a CO_2_ scrubber (e.g. soda lime, activated carbon, caustic soda) before reaching the detector. Additionally, water vapour can damage the laser sensors; hence, the gas is also passed through a water scrubber (e.g. calcium sulphate, calcium chloride, zeolites, silica gel) before reaching the detector. Many of the commercially available CO_2_ scrubber and desiccants contain indicator crystals that change colour as the scrubber absorb CO_2_ or water vapour, respectively. Another common sign when the scrubbers need to be replaced is the baseline signals shift significantly. To limit the frequency at which the scrubbers need to be replaced, longer scrubbing tubes could be used. However, this may increase the dead volume during measurements, as discussed later.

The measurement range of the ETD-300 depends on desired selected sensitivity, with possibility to switch between fine and coarse settings. The former is 100 times more sensitive that the latter, but the linearity between the photoacoustic signal of the laser and ethylene concentration is limited to 0–5 μL L^−1^. Conversely, when using the coarse settings, linearity extends to 500 μL L^−1^, allowing for measurements of much larger concentrations. For the purpose of this review, only the fine setting, with a theoretical measurement range of 0–5 μL L^−1^, will be considered. In many of the authors’ measurements, error can be up to 5 nL L^−1^, such that only measurements greater than 5 nL L^−1^ are often considered meaningful. When connected to one or more valve controllers, the ETD-300 can be operated in three modes: Continuous Flow, Stop and Flow, and Samples modes.

### Continuous Flow measurement

The Continuous Flow mode measures the steady state ethylene concentration of a sample. There is constant gas refreshment of all samples connected to the valve controller, hence avoiding the risk of CO_2_ accumulation that can influence plant physiology. The Continuous Flow method is recommended to be used if the sample produces sufficient ethylene. The period can be specified for each sample, and this determines how long the sample will be measured. In addition, the flow can be specified for each sample, and determines the rate at which the gas flows through the sample chamber.

#### Fruit ethylene production

Ethylene production is a common postharvest physiological measurement that can be obtained using the ETD-300 ethylene analyser. The valve control box controls mass flow rate and allows automatic sampling, making it possible to run several measurements in series (Fig. 1). More valve control boxes can be connected to the ETD-300, extending the possible number of simultaneous measurement channels by units of six with each control box. The baseline signal tends to drift with time, hence it is recommended to conduct a zero measurement before and after each measurement. The baseline value is obtained by measuring the signal from the ethylene-free carrier gas (channel 6, Fig. 1).

The maximum pressure of the carrier gas flowing to the catalyser is 6 atm. The ethylene-free gas from the catalyser is connected to a single input point in the valve control box, and is split internally into the different channels. The sample jar could be any sealed container that does not absorb or emit hydrocarbons. Flow of gas into each sample jar is achieved by positive pressure, controlled by the mass flow controller of valve control box. The mass flow controller in the valve control box measures the actual mass flow rate of the gas to the detector, which could be much lower than the set flow rate if the sample jar or connections are not airtight. Therefore, a good way to ensure sample is airtight is to check if the set flow is the same as the measured flow to the detector.

#### Time delay between measurements in Continuous Flow mode

At the start of a continuous mode experiment, a time delay exists as the system establishes an equilibrium state. A small delay is incurred between the initiation of measurement and when the gas from the first sample reaches the detector. This is easily established with knowledge of the applied flow rate and the free volume of the pipework. The vast majority of the time delay required is to enable equilibrium within the sample volume to be attained, and hence a constant ethylene concentration is observed. The free volume in the sample jar and the mass flow rate are two factors that influence this time delay. To calculate this time, a mass balance equation around the sample can be written as shown in Eq. (1), assuming that the gas in the sample jar is perfectly mixed.

1 $$V_{F} \frac{{d\left[ {C_{2} H_{4} } \right]_{out} }}{dt} = FR \times \left( {\left[ {C_{2} H_{4} } \right]_{in} - \left[ {C_{2} H_{4} } \right]_{out} } \right) + \phi_{{C_{2} H_{4} }} \times m$$

where $\left[ {C_{2} H_{4} } \right]_{in}$ (nL L^−1^) and $\left[ {C_{2} H_{4} } \right]_{out}$ (nL L^−1^) are the concentration of ethylene in gas flowing into and out of the sample, respectively, $FR$ (L h^−1^) is the flow rate, $V_{F}$ (L) is the free volume of the sample cuvette or jar (i.e., volume of cuvette–volume of plant material), *m* (kg) is the mass of the plant material, and $\phi_{{C_{2} H_{4} }}$ (nL kg^−1^ h^−1^) is the rate of ethylene production by the plant material. By substituting $\left[ {C_{2} H_{4} } \right]_{in}$ = 0, as the carrier gas is ethylene free (due to the catalyser), and rearranging Eq. (1), the following expression is obtained:

2 $$\frac{1}{{\phi_{{C_{2} H_{4} }} - FR \times \left[ {C_{2} H_{4} } \right]_{out} }}d\left[ {C_{2} H_{4} } \right]_{out} = \frac{1}{{V_{F} }}dt$$

If it is assumed that the ethylene production by the plant material is constant, Eq. (2) can be integrated from 0 to $\left[ {C_{2} H_{4} } \right]_{equil}$ (the equilibrium ethylene concentration defined as 0.95 × ${{\phi_{{C_{2} H_{4} }} } \mathord{\left/ {\vphantom {{\phi_{{C_{2} H_{4} }} } {FR}}} \right. \kern-0pt} {FR}}$) to obtain an expression for estimating the time delay, $\tau$ (h). It theoretically takes an infinite time to reach steady state. As a result, 95% equilibrium was selected as a close enough approximation for steady state.

3 $$\tau = - \frac{{V_{F} }}{FR}\ln \left( {0.05} \right)$$

**Fig. 2:**
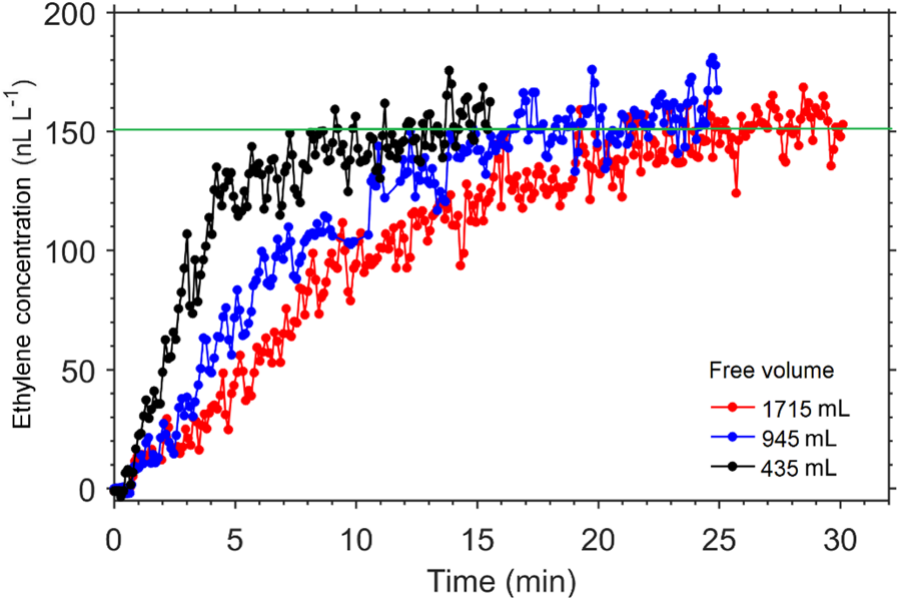
Real time ethylene from a single feijoa fruit (volume of 85 mL) measured by the ETD-300, using glass jars of different volumes, and a common flow rate of 5 L h^−1^. The time delay was defined as the time required to reach ~ 95% steady state (horizontal green line)

**Fig. 3:**
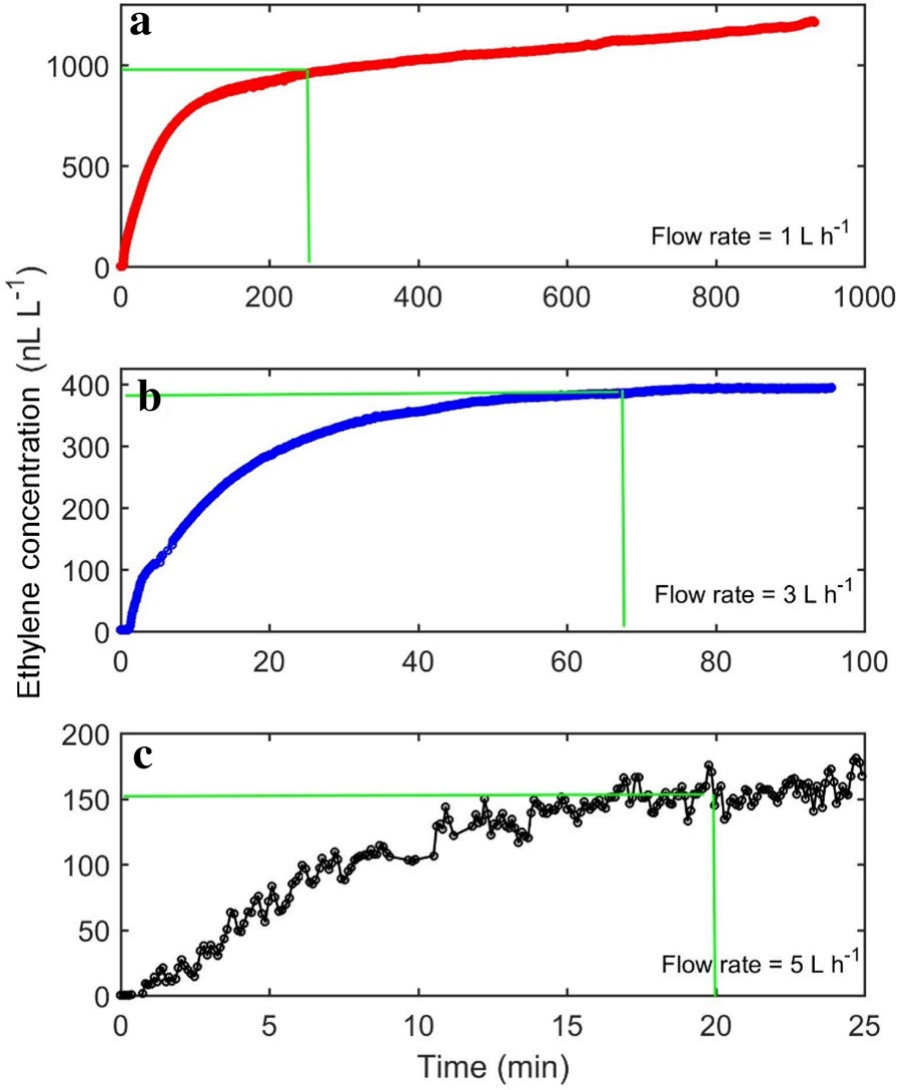
Real time ethylene from a single feijoa fruit (volume of 85 mL) measured by the ETD-300 in a 1000 mL glass jar at different flow rates (**a** 1 L h^−1^, **b** 3 L h^−1^, **c** 5 L h^−1^). Note differences in scale of axes. The time delay was defined as the time required to reach ~ 95% steady state (green lines). Note that A did not attain steady state

**Fig. 4:**
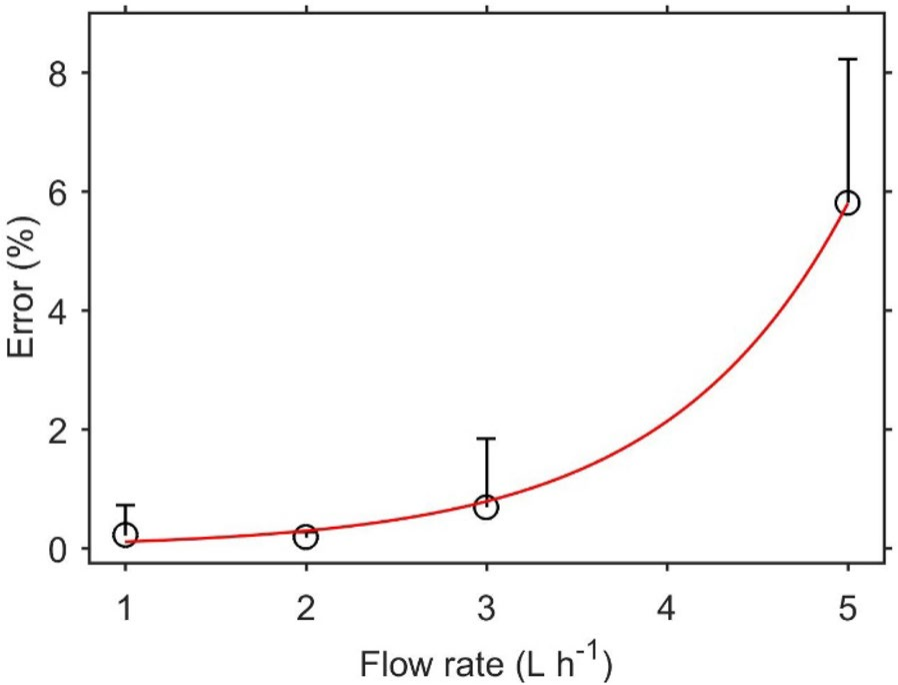
Error/noise to signal ratio resulting from using the Continuous Flow method at different flow rates to measure ethylene production of a 100 g feijoa fruit (producing 10 μL kg^−1^ h^−1^ ethylene). The error bars are the standard deviations of between 5 and 100 measurements

The effect of jar volume and flow rate on time delay was investigated by measuring ethylene from a single feijoa. Feijoa was used because of its relatively higher ethylene production rate, but with no physiological response to exogenous ethylene [34]. Time delay increases with increasing free space volume (Fig. 2), and decreases with increasing flow rate (Fig. 3). Using a combination of large free volume and small flow rate will result to extremely long time constant, and in some cases, equilibrium is never achieved (Fig. 3a), as the gas flow is unable to flush the ethylene being produced. This presents a risk of obtaining unreliable data, as ethylene production measurement in the continuous flow mode assumes constant ethylene production, which becomes less likely with longer periods, and the ethylene concentrations created in the system could stimulate changes in physiology. Generally, a high flow rate should be used for systems producing large amounts of ethylene, as this reduces the time delay considerably, and reduce the risk in breaking the assumption of constant ethylene production. As equilibrium ethylene concentration depends on the flow rate (Fig. 3), selecting very high flow rates may result to inability of the detector to measure the signal, as the resulting concentration may be below the sensitivity of the detector. In addition, higher flow rates means an increase in the error or noise to signal ratio (Fig. 4), and this is even more important if the equilibrium ethylene concentration is relatively low.

The effect of the length of the tubing connecting the sample jars to the valve control boxes and to the ETD-300 detector is often insignificant, as in most cases, the volume of the tubing is negligible when compared to that of the sample jars. For example, using a 2 m long tube with inner diameter of 2.22 mm will result to a tube volume of 7.7 mL, which is less than 2% of a small glass jar of 500 mL. The volume of the scrubbers could also add to overall volume. For example, the tubes the authors use to hold the CO_2_ and water vapour scrubbers have a volume of 30 mL and 60 mL, respectively. By assuming the scrubbers are packed to a porosity of ~ 15%, the additional volume by the scrubbing system is about 13.5 mL. This means the combined additional volume by the scrubbing system and the tubes is less than 5% of a free volume of 500 mL. Depending on the volume of the sample jar, the user may decide whether to consider this additional volume when applying Eq. (3) to estimate the time delay. If the time delay is too long (> 2 h), the free volume can be reduced by adding fillers into the jars, such as glass marbles. The amount of gas to be flushed is an attentive indicator of time delay (Fig. 2). When running several samples in series, the concept of time delay only applies to the first sample, as there is constant gas refreshment through all samples.

There are a number of practical ways to take into account the effect of the time delay during measurements. One way will be to set the first sample of the sequence to be a dummy sample that is programmed to run for the duration of the time delay. Alternatively, the first few samples could be repeated at the end of the sequence.

**Fig. 5:**
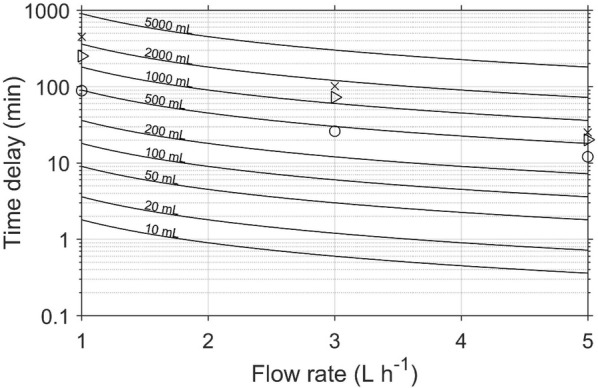
Effect of free space volume ($V_{F}$) and flow rate ($FR$) on time delay ($\tau$) when using the ETD-300 in the Continuous Flow mode. The points are time delays observed using continuous mode to measure ethylene of a single feijoa fruit (85 mL) in a 520 mL (×), 1000 (Δ), and 1800 mL (○) glass jars at mass flow rates of 1, 3, or 5 L h^−1^

To summarise the options for managing this time delay an operational chart for different cases of flow rates and free volume is provided (Fig. 5). To demonstrate that Eq. (3) provides a good estimate of the time delay, the observed time delays for different volumes and flow rate obtained by measuring ethylene production from a single ripe feijoa are also plotted in Fig. 5.

#### Selecting flow rate when using the Continuous Flow mode

When using the ETD-300 detector in the Continuous Flow mode, the concentration of ethylene in the gas reaching the detector is proportional to the ethylene production of the sample, and inversely proportion to the selected flow rate. This means for low ethylene producing samples a low flow rate should be selected, so that the ETD-300 detector can detect the concentration of ethylene produced by the sample. Contrarily, for a high ethylene-producing sample, a high flow rate should be used, such that the concentration of ethylene from the sample is not more than the maximum ethylene concentration that the detector can read. Beyond this concentration, the linearity of the photo-acoustic signal and ethylene concentration is not ascertained.

If the expected ethylene production, $\phi_{{C_{2} H_{4} }}$ (nL kg^−1^ h^−1^) of a sample is known, the concentration of ethylene, $C_{{C_{2} H_{4} }}$ (nL L^−1^), reaching the detector can be calculated for different flow rates, $FR$ (L h^−1^), using Eq. (4).

4 $$C_{{C_{2} H_{4} }} = m\frac{{\phi_{{C_{2} H_{4} }} }}{FR}$$

where $m$ (kg) is mass of the sample. If the ethylene production is reported in nmol kg^−1^ s^−1^, Eq. (5) can be used to estimate the concentration of ethylene reaching the detector.

5 $$C_{{C_{2} H_{4} }} = \frac{{m \times \phi_{{n,C_{2} H_{4} }} \times 3600 \times {\text{R}} \times T}}{FR \times P}$$

where $\phi_{{n,C_{2} H_{4} }}$ is the ethylene production in nmol kg^−1^ s^−1^, ${\text{R}}$ (8.314 L kPa mol^−1^ K^−1^) is the molar gas constant, $T$ (K) is temperature, and $P$ (kPa) is the atmospheric pressure.

**Fig. 6:**
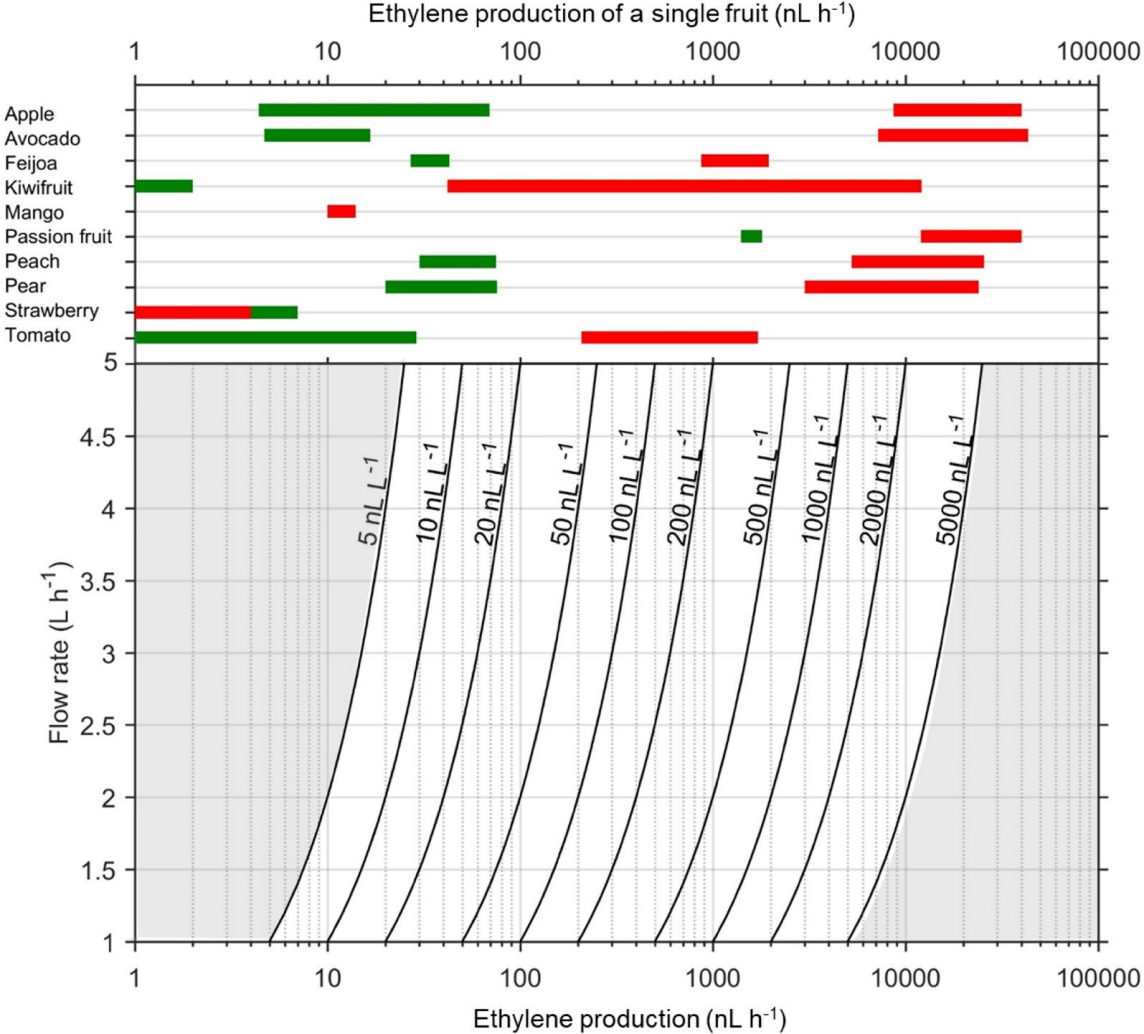
Guideline for selecting flow rate when using the ETD-300 detector in the continuous mode. The black solid curves are the concentrations of ethylene reaching the detector for different flow rates–ethylene production combinations, while the shaded area is outside the theoretical working range of the ETD-300. The green (unripe) and red (ripe) bars are the range of ethylene production of a single fruit

The range of ethylene production that can be measured by the ETD-300 detector operating in the Continuous Flow mode is 5–25,000 nL h^−1^. The lower limit was obtained by multiplying the minimum flow rate of 1 L h^−1^ by the lowest meaningful ethylene that can be detected (5 nL L^−1^), while the upper was obtained by multiplying the maximum flow rate of 5 L h^−1^ by the maximum ethylene that can be measured by the detector (5000 nL L^−1^). It is important to select the right flow rate and mass of fruit to get reasonable $\left[ {C_{2} H_{4} } \right]$ for measurement. Using Eq. (4), operational curves are drawn showing the concentration of ethylene reaching the detector as a function of flow rates and ethylene production of the sample (Fig. 6).

A survey of the postharvest literature was carried out to obtain ranges of ethylene production reported for different fruit at the unripe and ripe stages (Table 2). As an alternative to Table 2, users could refer to [42, 43] to obtain reasonable estimates of ethylene production for many fruit and vegetables. This data was used to estimate the range of ethylene production expected for a single fruit, and to suggest range of flow rates when measuring ethylene production of different fruit (Fig. 6). For example, a single unripe tomato produces ethylene at a rate of about 1–30 nL h^−1^, and a flow rate of about 1 L h^−1^ is recommended to measure production with the Continuous Flow method. Contrarily, for ripe tomato with an ethylene production of about 200–2000 nL h^−1^ for a single fruit, any flow rate between 1 and 5 L h^−1^ is suitable as the ETD-300 measurement will be between 50 and 500 nL L^−1^. If a high flow rate is used to measure a low ethylene producing fruit, the concentration of ethylene reaching the detector may be below the 5 nL L^−1^ practical lower limit of detection.

**Table 2:** Examples of expected range of ethylene production for some fruits

Fruit	Ethylene production (μL kg^−1^ h^−1^)		References
	Unripe	Ripe	
Apple	0.022–0.35	43.3–200	[44–47]
Avocado	0.026–0.095	40–240	[3, 7, 48]
Feijoa	0.27–0.43	8.7–19.5	[34, 35]
Kiwifruit	< 0.01	0.42–121	[40, 49, 50]
Mango	0.038–0.43	0.05–0.077	[36, 51]
Passionfruit	35–45	300–1000	[52, 53]
Peach	0.2–5.0	35–170	[54, 55]
Pear	0.1–0.38	15–120	[56–58]
Strawberry	0.095–0.580	0.003–0.346	[59, 60]
Tomato	0.012–0.48	3.46–28.6	[60–62]

For a number of climacteric fruit (e.g. kiwifruit, apple, avocado, peach, pear, and passion fruit) there is a log increase in ethylene production between the pre-climacteric and climacteric. Therefore, a change in measurement settings may be required when assessing fruit at different ripening stages. If no information is available about possible range of ethylene production for a sample, the highest flow rate of 5 L h^−1^ should be used, as this has the fastest response time, providing quick measurements for screening of right settings. Generally, 5 L h^−1^ is only suited for measurements of high ethylene production, as the noise to measurement ratio becomes less significant. For low ethylene producing fruits like unripe kiwifruit, mango, strawberry, the minimum flow rate of 1 L h^−1^ should be used.

As the ethylene production is proportional to the mass of fruit, increasing the amount of fruit is an option for increasing the ethylene production making it possible to measure samples that would have been otherwise undetectable. A point of consideration is that increasing the mass of fruit will ultimately require using sample jars of larger volume, thereby increasing the time delay (Fig. 3a) and introducing the risk of breaking the assumption of constant ethylene production. Another disadvantage of increasing mass of sample by measuring several fruit is that it takes away the true biological variance, and individual assessment of fruit can be important for ethylene production due to the log scale changes that occur during ripening. A single fruit may be producing over 100 times the ethylene of its neighbours, significantly skewing the average of the population.

#### Ethylene concentration in a local environment

**Fig. 7:**
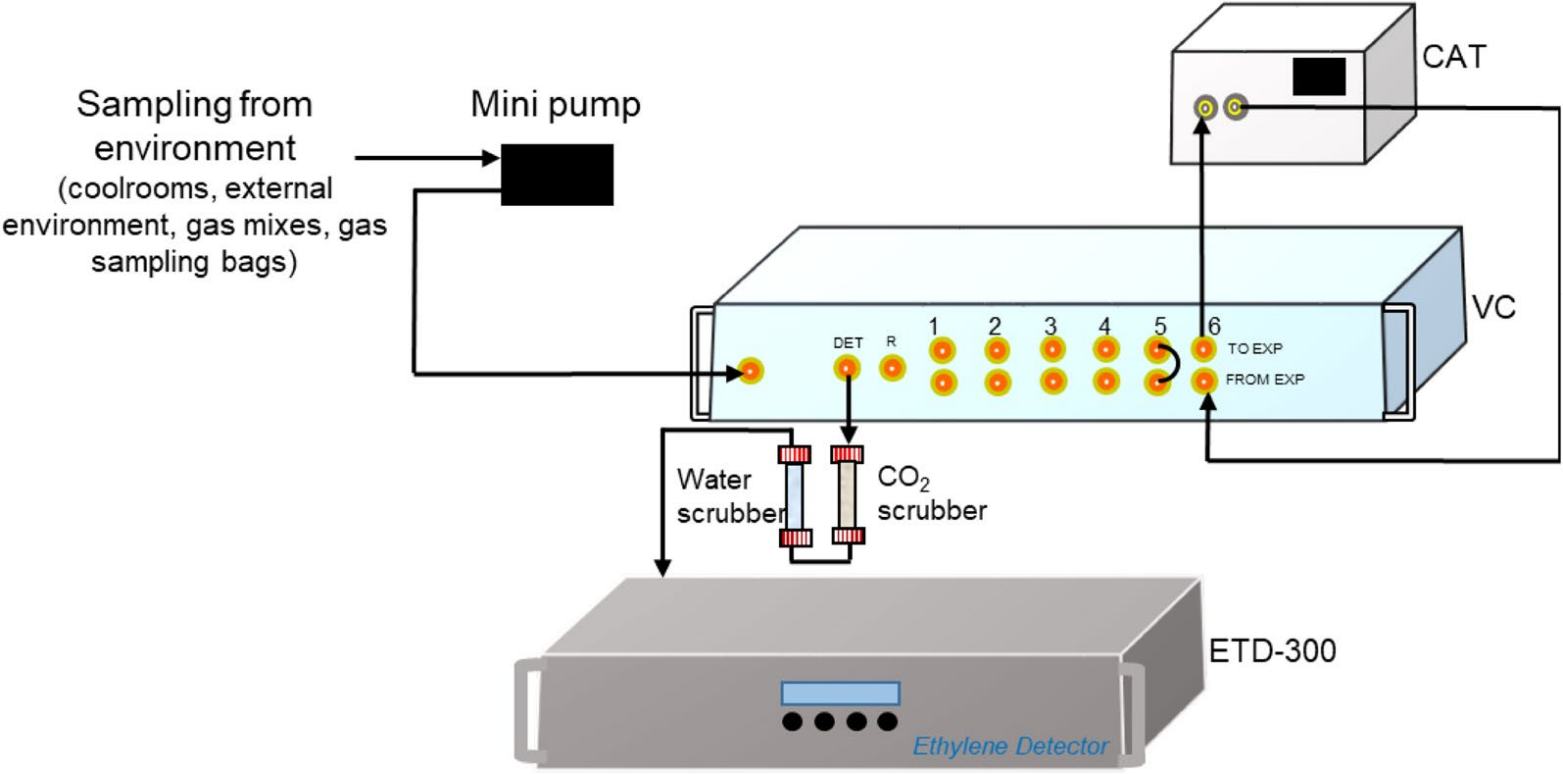
Setup for environmental monitoring

Although the ETD-300 was designed to measure ethylene production of confined samples, it can also be configured to monitor ethylene concentration of environmental conditions. To do this, a mini pump (e.g. LABPORT^®^, N 810 Pump; Aquarium Air Pump, RS-628A, and TCS micropumps, D10 K series) can be used to sample the gas from the environment of interest and provide the positive pressure required for the carrier gas (Fig. 7). This gas flow is directly feed to the control box rather than being scrubbed by the catalyser, using a simple loop in the valve controller (i.e., channel 5, Fig. 7) to enable the gas to be directed to the sensor. It remains important to have a zero baseline comparison, which can be achieved by passing the flow of another channel through the catalyser (i.e., channel 6, Fig. 7) before going to the ETD-300 detector.

Studies that investigate the effect of ethylene on plant response involve exposing plant material to different concentrations of ethylene (e.g. [3, 4, 7, 17, 18]). A common way to obtain different concentrations of ethylene gas is to mix ethylene standard gases with air by controlling the flow rates of the respective gases and sampling to verify if the desired ethylene concentration is achieved [17, 18, 63]. For mixes with low ethylene concentrations (< 5 μL L^−1^), the ETD-300 is suitable for checking the ethylene concentration in the mixed gas. To do this, the setup described in Fig. 7 can be used, with the pump connected to the sampling point of the mixed gas. The pump may not be necessary if there is gas flow at the sampling point, as the flow will provide the required positive pressure.

Sometimes it may be desirable to monitor ethylene concentration of a remote environment, such as in refrigerated containers or open market places. In this case, airtight sampling bags could be used to collect the gas sample from the remote environment and taken to location where the ETD-300 is installed. If the volume of the sample is much larger than the volume of the tubing system of the ETD-300, the ethylene concentration can be measured using the setup in Fig. 7, with the pump connected to the sampling bag. For small volumes (< 20 mL), the Samples method should be used (“Samples measurement” section).

#### Ethylene gas transport properties

Knowledge of gas permeability is an important design parameters when selecting films for packaging fruit, as this will influence the ethylene equilibrium within the package environment [64]. In addition, ethylene diffusion in fruit tissue may in part explain differences in response by different fruit. A common way to measure gas diffusion properties of a material is to use a system with two chambers separated by the sample whose diffusion properties is being measured [65–67]. A known concentration of the gas of interest is flushed through one of the chambers, while the concentration of the gas in the other chamber is continuously monitored. The ability of the ETD-300 to measure real time ethylene, and both control and measure flow rate, makes it suitable for measuring ethylene diffusion properties. Moreover, the low detection limit of the ETD-300 means small increases in ethylene in the measurement chamber could be accurately measured.

**Fig. 8:**
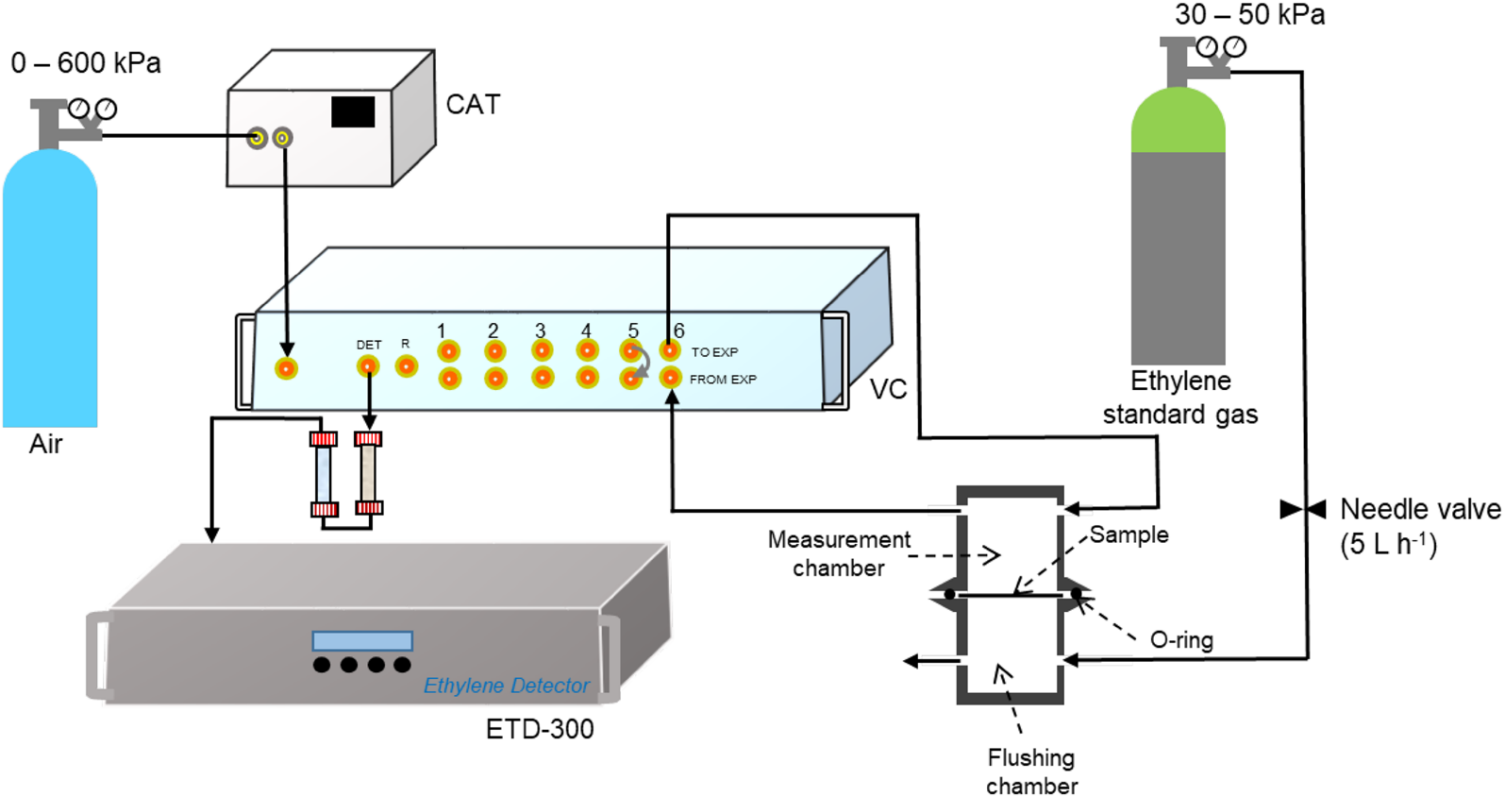
A schematic of ETD-300 set for measuring ethylene diffusion properties of membrane using a diffusion chamber

The ETD-300 could be used to measure ethylene diffusion properties of films and plant tissue using the set up shown in Fig. 8. Ethylene of a known concentration is passed through the flushing chamber, while the inlet and the outlet of the measurement chamber is connected to the ETD-300 via the valve control box (channel 6, Fig. 8). The ethylene diffusion properties of the sample is estimated by fitting the measured real time ethylene data to gas transport equations (Eq. (6)).

6 $$\frac{{dC_{{in,C_{2} H_{4} }} }}{dt} = \frac{{P_{{C_{2} H_{4} }} \times d}}{A}\left( {C_{{out,C_{2} H_{4} }} - C_{{in,C_{2} H_{4} }} } \right)$$

where $C_{{in,C_{2} H_{4} }}$ (nL L^−1^) and $C_{{out,C_{2} H_{4} }}$ (nL L^−1^) are the ethylene concentrations in the measurement and flushing chambers, respectively, $P_{{C_{2} H_{4} }}$ (m^3^ m^−2^ s^−1^) is the ethylene permeance of the membrane, $A$ (m^2^) is the cross sectional area, and $d$ (m) is the thickness of the membrane.

### Stop and Flow measurement

The Stop and Flow method of measurement can be used either for samples that produce very low amounts of ethylene, or for small samples collected remotely from the equipment in a sealed vessel. In the Stop and Flow mode, the complete volume of gas in the free space of the sample is measured, producing a peak from which the amount of ethylene can be calculated from the area under the peak. The main disadvantage of this method when measuring ethylene production is that CO_2_ or other volatiles can accumulate to levels that may affect physiological processes. As with the Continuous Flow mode, the period and the flow rate for each sample can be specified. The period determines how long the sample will be measured, while the flow rates determine the rate at which gas flows from the sample to the detector. It is important to flush the system by running ethylene-free gas before the start of measurement, as this ensures the system does not contain any residual ethylene from previous samples, and that the peak starts from the true baseline.

#### Fruit ethylene production

If ethylene production of a sample is less than 10 nL h^−1^, the concentration of ethylene reaching the detector during measurement in the Continuous Flow mode at a flow rate of 1 L h^−1^ could be just above the lower measurement limit of 5 nL L^−1^ (Fig. 5). In such situations, it would be more accurate to measure ethylene production using the Stop and Flow method. The setup in Fig. 1 is used to measure ethylene production of a sample. The output generated is an ethylene concentration peak, from which the total amount of ethylene is obtained as the area under the curve (nL). The ethylene production (nL h^−1^ kg^−1^) is obtained by dividing the amount of ethylene by the residence time and the mass of the sample.

#### Residence time when using the Stop and Flow mode

For ethylene production measurement, the samples are confined in sealed jars, allowing accumulation of ethylene produced by the plant material. A practical consideration is the length of time to allow for ethylene accumulation. If this is too long, CO_2_ may accumulate to concentrations that will affect the product physiology, negating the assumption of constant physiology. If too short, the plant material would not produce enough ethylene that is detectable by the ETD-300.

To calculate the minimum residence time, $t_{\text{res}}$ (h), let us consider that the detection limit of the ETD-300 is $\left[ {C_{2} H_{4} } \right]_{\text{limit}}$ (nL L^−1^). If $\phi_{{C_{2} H_{4} }}$ (nL kg^−1^ h^−1^) is the ethylene production of the sample (e.g. Table 2), then the minimum residence time can be calculated using Eq. (7).

7 $$t_{\text{res}} = \frac{{\left[ {C_{2} H_{4} } \right]_{\text{limit}} \times V_{F} }}{{m \times \phi_{{C_{2} H_{4} }} }}$$

If the ethylene production is reported in nmol kg^−1^ s^−1^, the minimum residence time can be calculated using Eq. (8).

8 $$t_{\text{res}} = \frac{{\left[ {C_{2} H_{4} } \right]_{\text{limit}} \times V_{F} \times P}}{{m \times \phi_{{n,C_{2} H_{4} }} \times 3600 \times {\text{R}} \times T}}$$

**Fig. 9:**
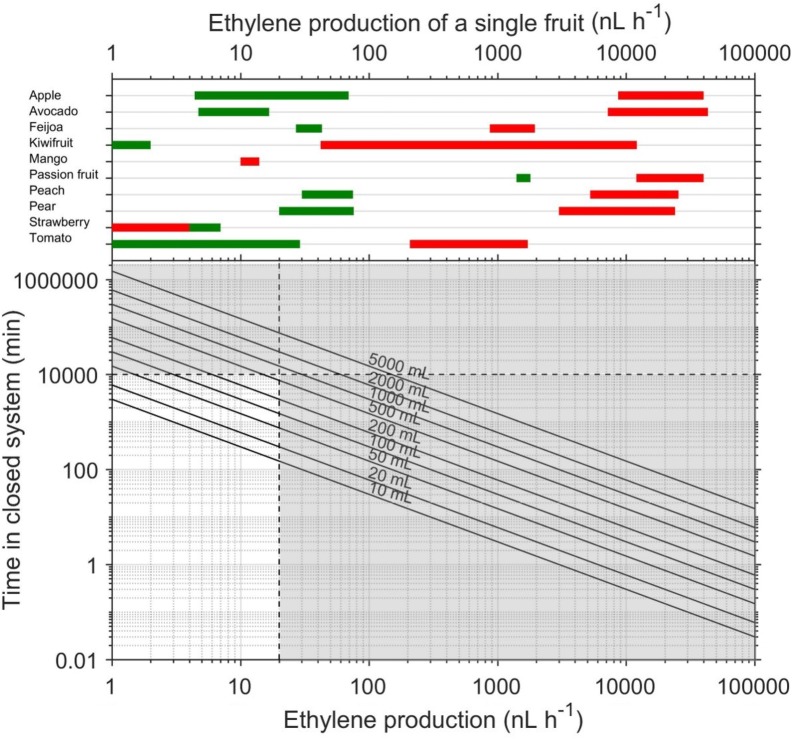
Effect of free volume of sample jar and ethylene production on the minimum residence time in a closed system to allow for detectable level of ethylene (5 nL L^−1^). The black solid diagonal lines are for a given free volume, while the shaded area is outside the practical range. The green (unripe) and red (ripe) bars are the range of ethylene production of a single fruit

Using Eq. (7), and assuming the minimum detectable ethylene ($\left[ {C_{2} H_{4} } \right]_{\text{limit}}$) is 5 nL L^−1^, the effect of free volume ($V_{F}$) and ethylene production ($\phi_{{C_{2} H_{4} }}$) of the sample on the minimum residence time is shown in Fig. 9.

Before using the Stop and Flow mode of the ETD-300 detector to measure ethylene production of a plant material, the researcher should first consult the literature to know what values of ethylene production is expected for the given sample. When this is known, Eq. (7) could be used to estimate the minimum residence time. Operational curves of the minimum residence time to allow for accumulation of 5 nL L^−1^ ethylene when measuring produce in a closed system as a function of free volume and ethylene production is shown in Fig. 9. Using expected range of ethylene production of a single fruit for different fruit categories (Table 2), suggested residence time for measuring single fruit in closed system with different free volume is also shown on Fig. 9. Measurement of samples with ethylene production less than 20 nL h^−1^ requires over 7 days, if the free volume is more than 200 mL. Using more fruit will increase ethylene production, while decreasing the free volume, thereby reducing the time required to produce a detectable amount of ethylene. For example, if ethylene production of ripe strawberries is measured using five fruit (~ 60 mL) in a 500 mL jar, the minimum residence time in the closed system will be less 1 h. The ethylene production of similar low ethylene producing fruit and non-climacteric fruit, such as citrus, could be measured using a combination of increasing mass of sample and using longer residence time. Because samples with ethylene production of more than 20 nL h^−1^ can easily be measured with the Continuous Flow method (Fig. 5), the Stop and Flow method has a narrow practical working range (unshaded area in Fig. 9). This narrow range is mainly for samples with small volumes (10–200 mL), which is a rare case when measuring fruit as most fruit will only fit into at least 500 mL jars, and hence may explain why the ETD-300 is rarely used in the Stop and Flow mode during measurement of ethylene production in postharvest biology. With the exception of Anastasiadi et al. [37], all the references discussed in the introduction used the ETD-300 in the Continuous Flow mode.

When using Stop and Flow method to measure plant samples, users need to consider an additional risk of CO_2_ accumulation if the produce is left for too long within the closed system. If the rate of respiration is known, the following expression can be used to estimate CO_2_ accumulation, $C_{{{\text{CO}}_{ 2} }}$ (%), as a function of residence time within a closed system:

9 $$C_{{{\text{CO}}_{ 2} }} = \frac{{m \times RR_{{{\text{CO}}_{ 2} }} \times t_{res} \times 10^{ - 7} }}{{V_{F} }}$$

where $RR_{{{\text{CO}}_{ 2} }}$ (nL kg^−1^ h^−1^) is the respiration rate in terms of CO_2_ accumulation, and 10^−7^ provides unit conversion from part per billion (or nL L^−1^) to percent. Assuming that a measurement of ethylene production is being conducted in air, during the experiment a tolerance limit of 0.5% CO_2_ may be applied. The ratio of CO_2_ production to ethylene production determines whether CO_2_ will accumulate to this tolerance limit before production of detectable ethylene. If we assume an ETD-300 detection limit of 5 nL L^−1^ and a CO_2_ tolerance limit of 0.5%, a point at which physiology starts to be influenced for many crops [68], combining Eqs. (7) and (9) provides the following expression for estimating a risk of CO_2_ accumulation during Stop and Flow measurement:

10 $$\frac{{RR_{{{\text{CO}}_{ 2} }} }}{{\phi_{{C_{2} H_{4} }} }} > 10^{6}$$

**Fig. 10:**
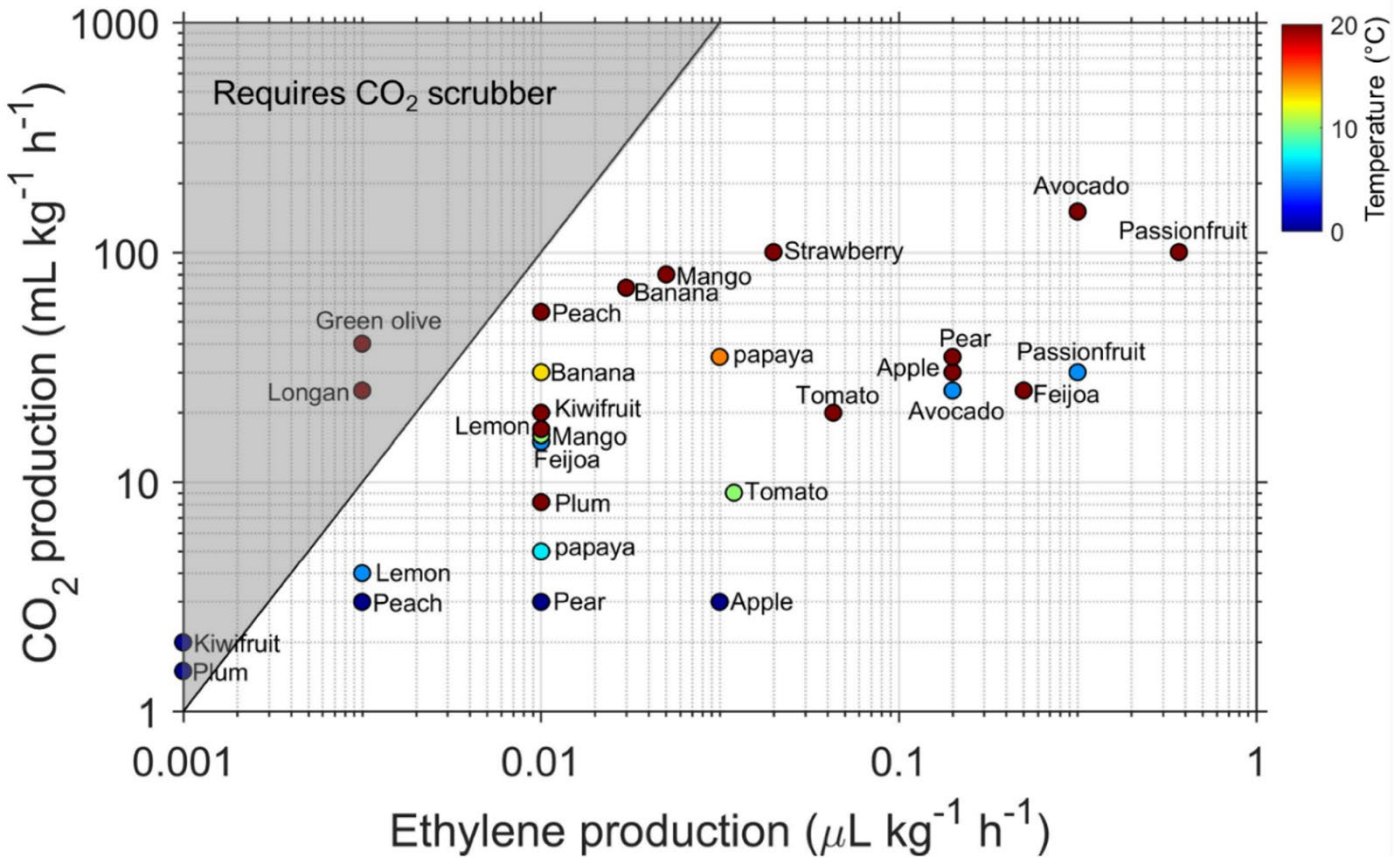
Relationship between ethylene production and CO_2_ production for different fruit at different temperatures (data obtained from UC David Produce Facts). The line represents CO_2_ production = 10^6^ × ethylene production, which defines the point above which CO_2_ will accumulate to > 0.5%

Based on this expression, if $RR_{{{\text{CO}}_{ 2} }}$ > $10^{6} \times \phi_{{C_{2} H_{4} }}$, then there is a potential problem of CO_2_ during measurement, and therefore a CO_2_ scrubber is needed during Stop and Flow accumulation (shaded region in Fig. 10). To verify the risk of CO_2_ accumulation during ethylene measurements of different fruit with the Stop and Flow method, ethylene production was plotted against CO_2_ production (Fig. 10) based on data reported in the UC Davis Produce Facts. A conservative approach was adopted by using the lower value for the range of ethylene production and the upper value for the range of CO_2_ production data at 0–20 °C. During measurement of ethylene production in a closed system, the risk of CO_2_ accumulation is much lower for fruit that have both high rates of respiration and ethylene production (such as avocado, passion fruit, Feijoa, apples), as a relatively short residence time is needed for detectable ethylene to be produced. In contrast, fruit such as unripe kiwifruit and plum that produces very low ethylene, but with relatively high respiration rates poses a risk of CO_2_ accumulation during measurements in a closed system. This is because measurement of ethylene production of such fruit requires long residence time, increasing the risk of CO_2_ accumulation. For example, it will take theoretically about 3 months to produce detectable ethylene when measuring ethylene production of a single unripe kiwifruit in a closed system with free volume of 1 L, while it will take the same fruit about 2–3 h to accumulate 0.5% CO_2_. Having more fruit in the jar will reduce the residence time for accumulation of detectable ethylene, but will also proportionately increase the rate of CO_2_ accumulation. A number of fruit occupy the region just below the critical line of CO_2_ risk (Fig. 10; Peach, Banana, Lemon), meaning if the fruit are left in a closed system for a much longer time than the minimum residence time, CO_2_ may accumulate to more than 0.5%.

CO_2_ production rate is highly dependent on ripening stage, and on environmental conditions (temperature, and gas composition), such that the most practical way to ensure CO_2_ is kept below critical level for samples needing relatively long residence time is to have a septum on the jar for periodic sampling of small gas for CO_2_ measurements. Alternatively, CO_2_ scrubbers could be placed inside the closed system to limit accumulation.

#### Selecting flow rate during Stop and Flow measurement

**Fig. 11:**
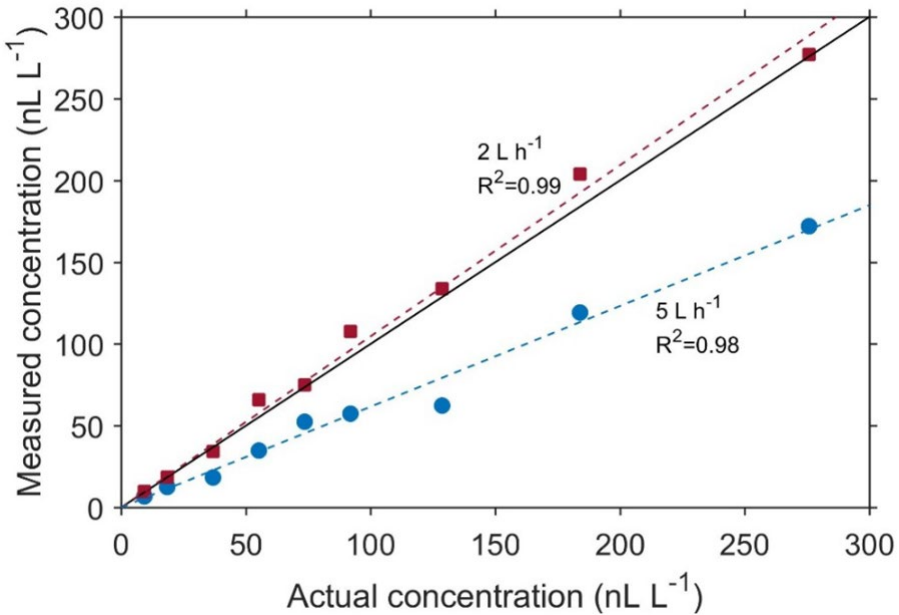
Comparison between measured ethylene when flushing a 520 mL glass jar at a flow rate of 2 or 5 L h^−1^. The black solid line is the ideal correlation

Using the highest possible flow rate of 5 L h^−1^ is theoretically the most appropriate choice during Stop and Flow measurement, as this will require the least amount of time to completely flush the sample. To investigate if flow rates influence measurement accuracy, different ethylene concentrations were generated by injecting different volumes of 0.96 μL L^−1^ ethylene standard gas into 520 mL jars. The ethylene concentration in the jars were measured using the ETD-300 operating in the Stop and Flow mode, with a flow rate of either 2 or 5 L h^−1^ (Fig. 11).

**Fig. 12:**
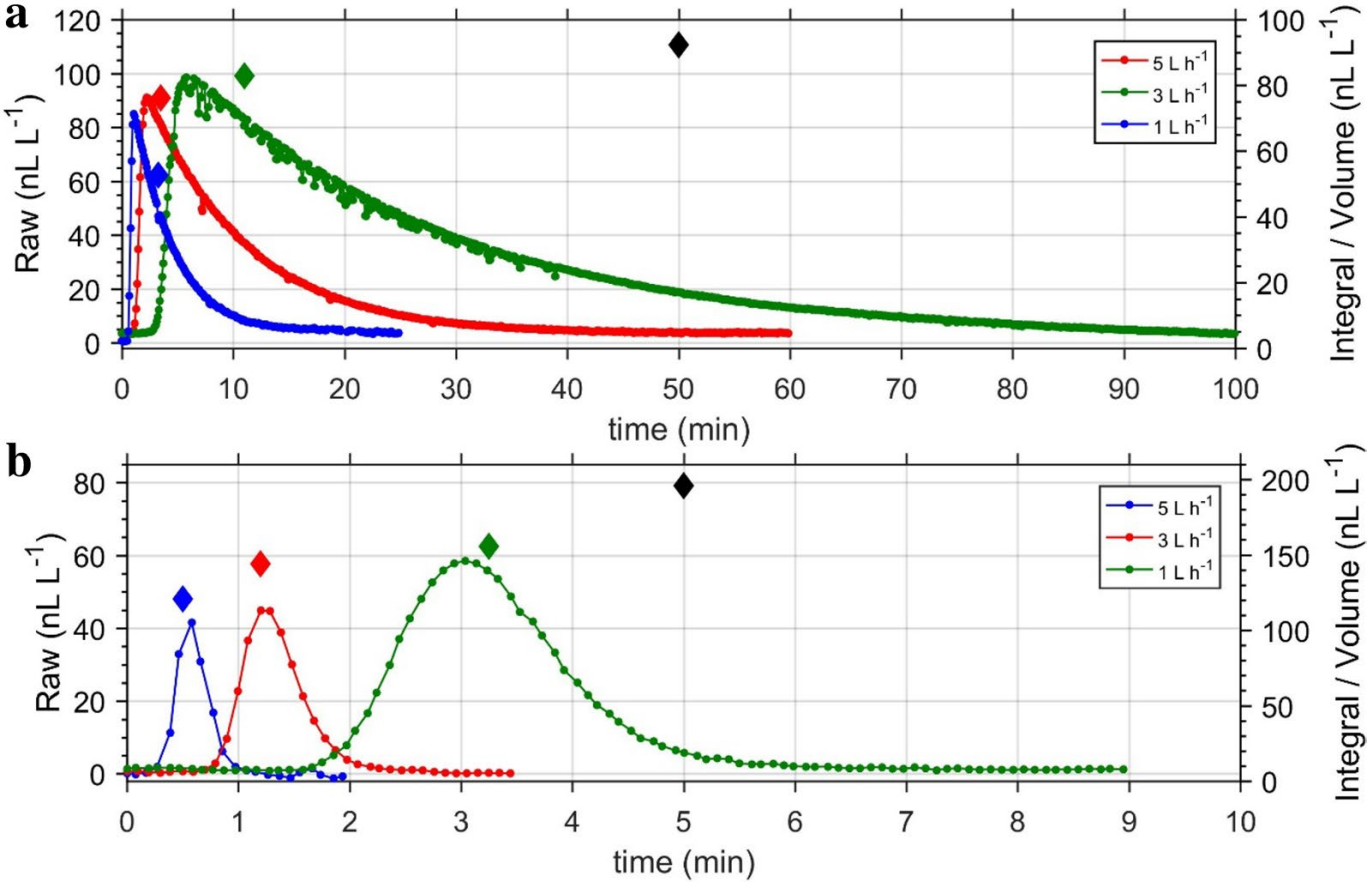
Effect of flow rates and sample volume on Stop and Flow measurements. The ethylene concentration curves were obtained from using the Stop and Flow method at different flow rates to measure the same concentration of ethylene gas in a large volume (196 nL L^−1^ in 520 mL; **a**) or a small (92.31 nL L^−1^ in 10 mL; **b**). The coloured diamond are the measured concentrations using the respective flow rates, while the black diamond is the actual concentration

Although the measured ethylene concentration at both 2 and 5 L h^−1^ were correlated to the actual concentrations, using 5 L h^−1^ resulted to systematic underestimation of the ethylene concentration. To investigate this further, we prepared identical concentration of ethylene in three 520 mL glass jars and measured the concentration with the Stop and Flow method, using flow rates of 1, 3, or 5 L h^−1^ (Fig. 12a). The experiment was repeated with a smaller volume (10 mL; Fig. 12b). For both experiments, using 5 L h^−1^ resulted to rapid flushing of the gas sample, causing a significant underestimation of the amount of ethylene. The accuracy of the measured ethylene concentration profile during the Stop and Flow method is a function of the measurement speed (which is 5 s for the ETD-300) to gas flow ratio. If this ratio is too low (in the case of using 5 L h^−1^), the error in the ethylene concentration profile is considerably high, as some of the gas is flushed through the detector without being measured. Based on this result, we recommend using 1–2 L h^−1^ during Stop and Flow measurements. The effect of flow rate on the accuracy of measurement is minimal for the Continuous Flow method, as the concentration is, in principle, constant during measurement.

#### Effect of cuvette volume on ETD-300 performance when using the Stop and Flow mode

**Fig. 13:**
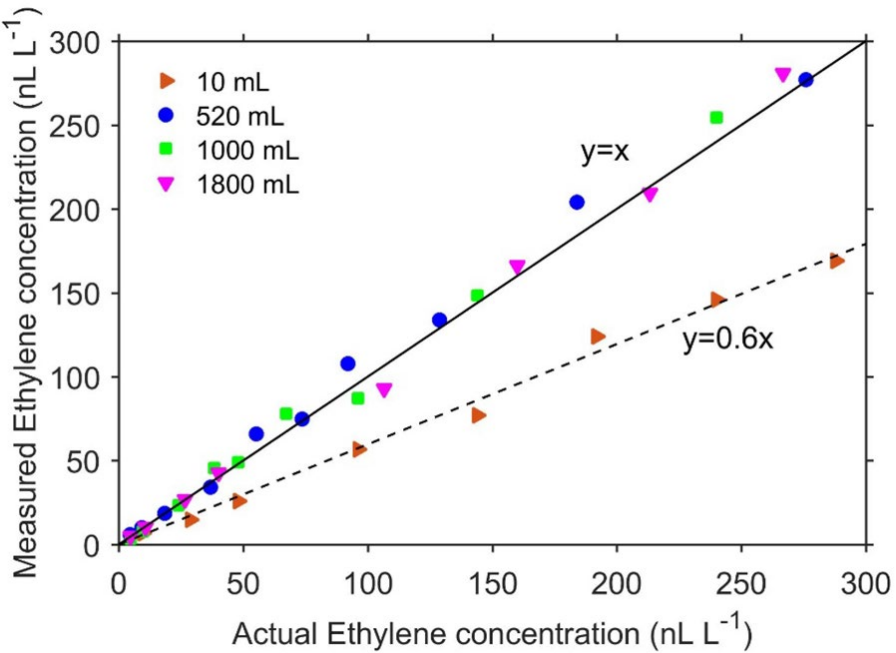
Correlation between actual ethylene and ethylene measured by the ETD-300 operating in the Stop and Flow mode for different jar volumes

When running the ETD-300 in the Stop and Flow mode, using sample jars with large volumes results in broad peaks (Fig. 12a), as a much longer time will be required to completely flush the gas within the sample. In this case, the period (in addition to the residence time) needs to be taken into account when estimating ethylene production rate, as additional ethylene would have been produced during this time. Initial observation from Fig. 12 suggests the sample free volume has an effect on the measured concentration, with smaller sample volumes (10 mL compared to 520 mL) resulting in underestimation of the ethylene concentration. To further investigate if the measured concentration when using the Stop and Flow method is a function of the free volume of the sample, different ethylene concentrations were generated in 10, 520, 1000, and 1800 mL jars by injecting different volumes of 0.96 μL L^−1^ ethylene standard gas. The ethylene concentration in the respective jars were measured with the ETD-300 operating in the Stop and Flow mode, using a flow rate of 2 L h^−1^ (Fig. 13). The measured ethylene concentration for free volume of 520, 1000 and 1800 mL were comparable, and closed to the actual concentrations. However, using the 10 mL gas sample, the measured ethylene was lower than actual ethylene concentration. This means when smaller sample volumes are measured using the Stop and Flow method, there is risk in underestimating the actual ethylene concentration. As earlier explained, an error related to the measurement speed to gas flow is associated with estimating the ethylene concentration profile when using the Stop and Flow method. This error is not only dependent on gas flow rates during measurement, but on volume of sample gas with the error being more significant if only small volume of gas is measured. For larger volumes and low gas flow rates, this error tends to be minimal (Fig. 12a, 1 L h^−1^ flow). Most measurements of ethylene production of fruit and vegetables will have free volumes of at least 500 mL. When using the Stop and Flow method to measure ethylene production of other plant materials using small volume cuvettes, this discrepancy between measured and actual ethylene should be taking into account.

#### Selecting period during Stop and Flow measurements

**Fig. 14:**
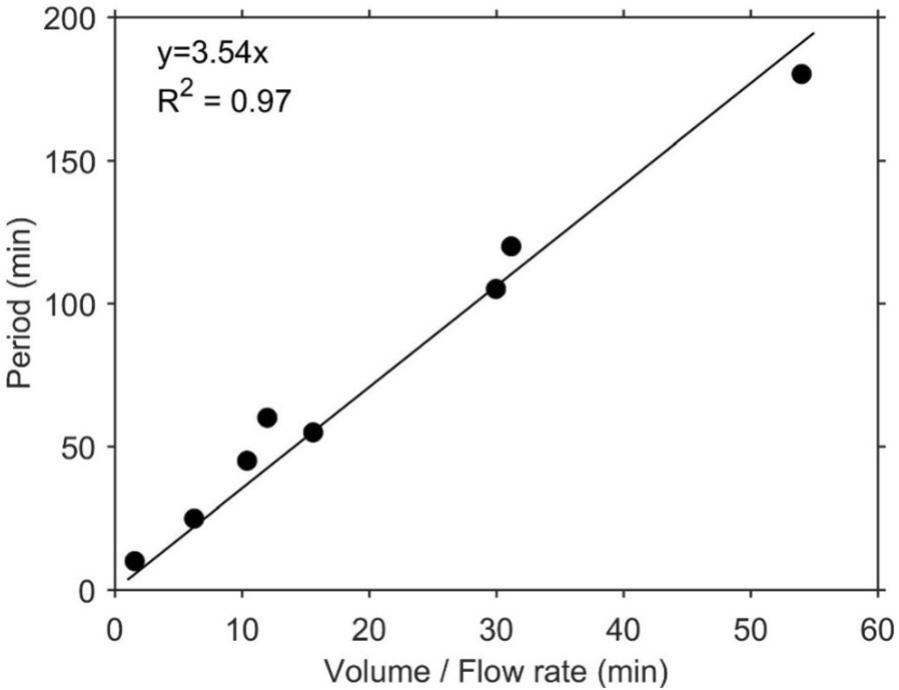
Relationship between free volume ($V_{F}$) to flow rate ($FR$) ratio and period during Stop and Flow measurements

A period needs to be selected such that there is complete flushing of the sample, indicated by a return of the ethylene measurement signals to the constant baseline level. The period depends on the selected flow rate and the free volume of sample, with higher flow rates requiring shorter periods, and larger volumes requiring longer periods. To quantify this relationship, we determined the time required to flush different volume of sample at different flow rates. A linear relationship was obtained between the volume to flow rate ratio (i.e. time needed to flush a single volume) and the period, with a regression coefficient of 3.54 (Fig. 14). This means a rule of thumb for estimating the minimum period for a Stop and Flow measurement is to calculate the time needed to flush four volumes of the sample.

### Samples measurement

The Sample method measures the ethylene concentration of a sample by comparing to reference samples. Both the Continuous Flow and the Stop and Flow methods are useful when it is desirable to measure the ethylene production over time, while the Samples mode of measurement is used when samples need to be analysed only once. For example, measuring the ethylene concentration of a gas sample collected remotely, or measuring ethylene production of produce after storage. The equipment configuration and measurement output are similar to the Stop and Flow mode, with the main difference being that the Samples mode compares the reference samples (standards) with other samples. This implies the flow and the gas volume of all samples measured in series need to be identical. The ethylene concentration of the reference samples has to be provided, and is used to generate a calibration line between the peak area and the concentration.

#### Measuring ethylene concentration of a small volume

The error in measured ethylene concentration when using the Stop and Flow method to measure samples with small free volumes can be circumvented by using the Samples method, as this uses the concentration of reference samples to estimate the concentration of the samples. This incorporates the error associated with the sampling method (free volume, flow rate, change in pressure due to injection of the gas into the cuvette) into the measurements. It is important that the reference cuvettes are prepared and measured in the same way as the sample, i.e. the cuvette volume, volume of sample gas injected, measurement flow rate and period.

**Fig. 15:**
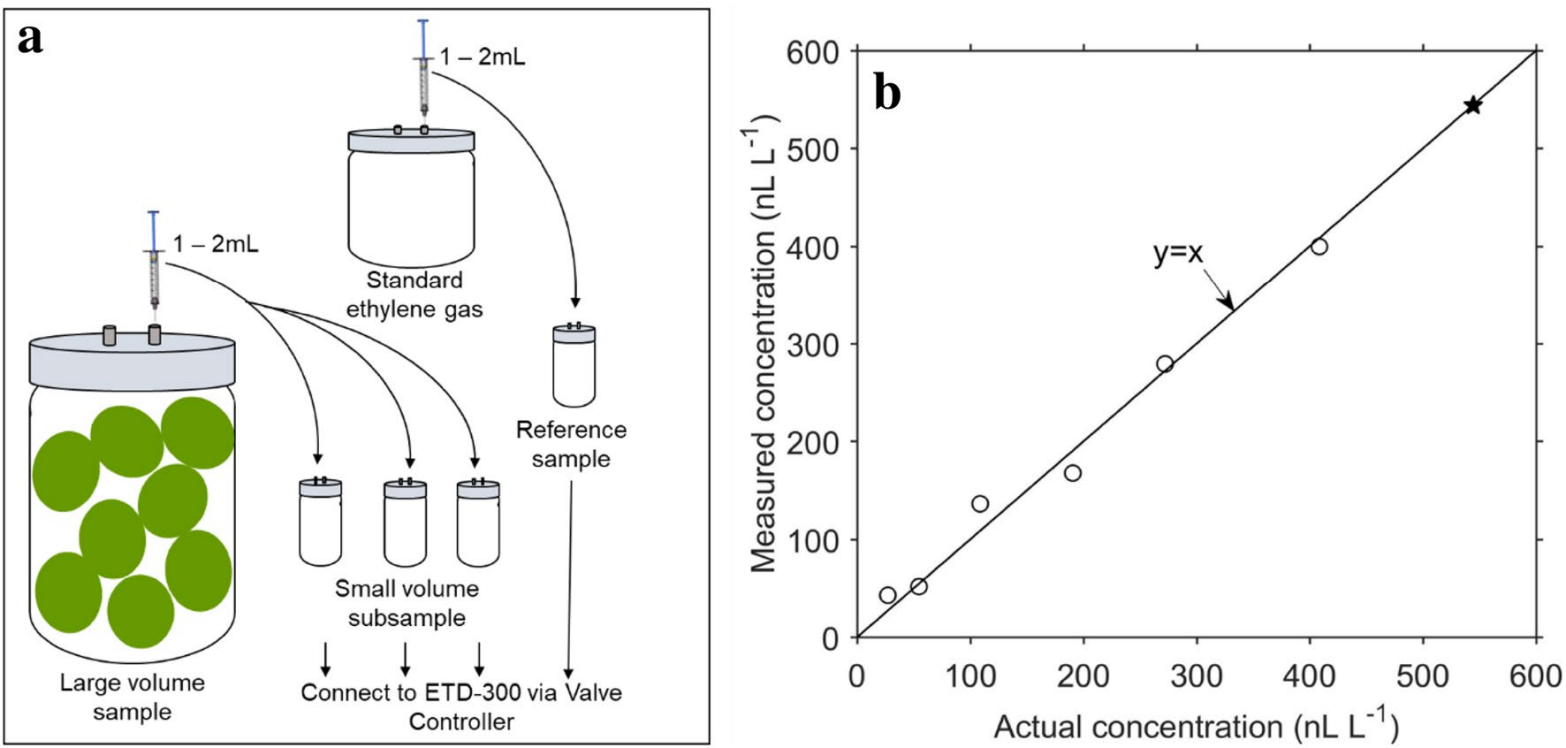
Using the Samples method to measure small volume subsamples from larger volume samples by taking a 1–2 mL subsample gas from larger sample and injecting into smaller cuvettes (**a**). As an example, different concentration of ethylene in 1800 mL glass jars was measured by injecting 2 mL of sample gas into 10 mL cuvettes (**b**). The black filled pentagram is the measurement of the reference cuvette

A practical limitation of the Stop and Flow method for samples with large free volume is that the measurement requires very long periods. This increases the risk of CO_2_ accumulation, and the risk of breaking the assumption of constant ethylene production. To avoid this, a small gas volume (1–2 mL) can subsampled from the larger sample, injected into a smaller cuvette and measured using the Samples method (Fig. 15). In the example shown in Fig. 15, the best correlation between the measured and actual concentration was obtained when the highest concentration sample was used as reference. Consequently, we recommend using high concentrations (> 200 nL L^−1^) for the reference samples, as the error in measurement is smaller with higher concentrations. In addition, after injecting the subsample in the small cuvette, sufficient mixing (by shaking the tube and leaving to sit for a few minutes) to ensure perfect mixing is needed before measurement.

Another situation where it may be desirable to measure small volumes is monitoring ethylene concentration of an environment remote to the ETD-300 equipment (e.g. during transport). In this case, using airtight containers, small volumes of gas sample can be collected from the remote environment, and later measured using the Samples method. Even when large volumes of gas are collected, a small subsample can be analysed in smaller cuvettes, as described in the previous paragraph.

## Conclusion

**Fig. 16:**
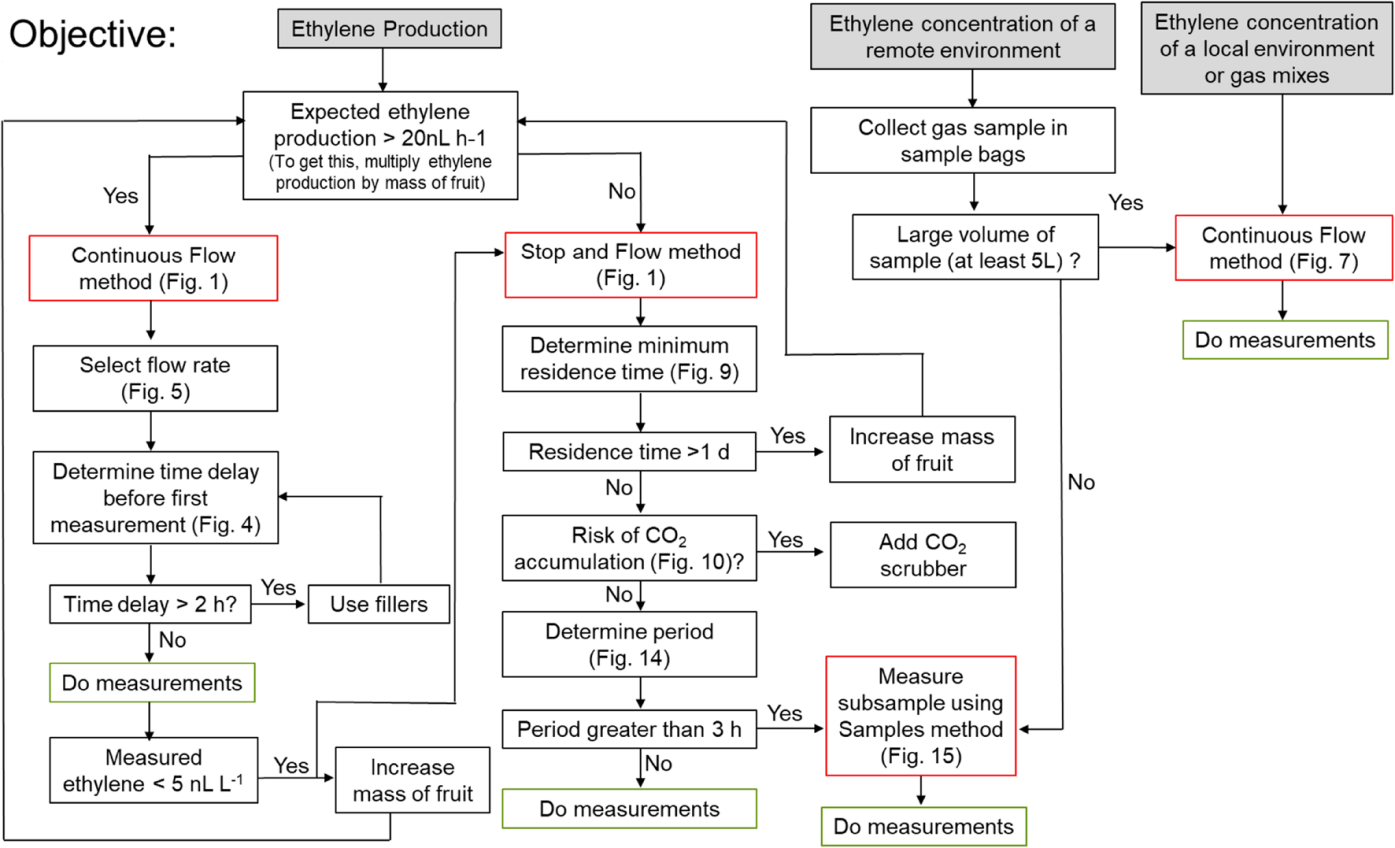
Decision diagram when using the ETD-300 for different type objectives (ethylene production, ethylene concentration of a remote environment, and ethylene gas concentration of a local environment or gas mixes). The red squares indicate the different types of measurements, while the green squares indicate at what point you should proceed with measurements

The laser-based photoacoustic ethylene detector ETD-300 allows quantification of ethylene concentrations at higher sensitivities than all current ethylene-sensing technologies. Additionally, the equipment is able to monitor ethylene in real time. Being a relatively new piece of equipment, there is considerable learning needed for its optimal usage. The three modes of measurements (Continuous Flow, Stop and Flow, and Sample measurements) all have their unique advantages and are suited for different measurements (Fig. 16). The Continuous Flow method is useful for measuring high ethylene producing samples. The expected ethylene production should be used as a guide for selecting the flow rates. A time delay is always associated with measurement using the Continuous Flow method. In addition to measuring ethylene production, the setup of the ETD-300 can be adapted to allow for monitoring of environment in the Continuous Flow mode. The Stop and Flow method is suitable when measuring low ethylene production, by allowing time for enough ethylene to accumulate in a closed system. For large volumes, very long periods are required to completely flush the gas sample in Stop and Flow measurement. The Sample method is useful for measurement of small volumes.
